# Seawater softening of suture zones inhibits fracture propagation in Antarctic ice shelves

**DOI:** 10.1038/s41467-019-13539-x

**Published:** 2019-12-02

**Authors:** Bernd Kulessa, Adam D. Booth, Martin O’Leary, Daniel McGrath, Edward C. King, Adrian J. Luckman, Paul R. Holland, Daniela Jansen, Suzanne L. Bevan, Sarah S. Thompson, Bryn Hubbard

**Affiliations:** 1 Glaciology Group, College of Science, Swansea University, Singleton Park, Swansea, SA2 8PP UK; 2 School of Technology, Environments and Design, University of Tasmania, Hobart, TAS 7001 Australia; 3 Institute of Applied Geoscience, School of Earth and Environment, University of Leeds, Leeds, LS2 9JT UK; 4 Department of Geosciences, Colorado State University, Fort Collins, CO 80523-1036 USA; 5 British Antarctic Survey, Natural Environment Research Council, Madingley Road, Cambridge, CB3 0ET UK; 6 Centre for Glaciology, Department of Geography and Earth Sciences, Aberystwyth University, Aberystwyth, SY23 3DB UK; 7 Present Address: Division of Glaciology, Alfred-Wegener Institute for Polar and Marine Research, 27568 Bremerhaven, Germany; 8 Present Address: Institute for Marine and Antarctic Studies, University of Tasmania, Hobart, TAS 7001 Australia

**Keywords:** Climate change, Cryospheric science

## Abstract

Suture zones are abundant on Antarctic ice shelves and widely observed to impede fracture propagation, greatly enhancing ice-shelf stability. Using seismic and radar observations on the Larsen C Ice Shelf of the Antarctic Peninsula, we confirm that such zones are highly heterogeneous, consisting of multiple meteoric and marine ice bodies of diverse provenance fused together. Here we demonstrate that fracture detainment is predominantly controlled by enhanced seawater content in suture zones, rather than by enhanced temperature as previously thought. We show that interstitial seawater can reduce fracture-driving stress by orders of magnitude, promoting both viscous relaxation and the development of micro cracks, the incidence of which scales inversely with stress intensity. We show how simple analysis of viscous buckles in ice-penetrating radar data can quantify the seawater content of suture zones and their modification of the ice-shelf’s stress regime. By limiting fracture, enhancing stability and restraining continental ice discharge into the ocean, suture zones act as vital regulators of Antarctic mass balance.

## Abstract (web summary)

Suture zones are abundant on Antarctic ice shelves and widely observed to impede fracture propagation. Here we show that fracture detainment is principally controlled by the zones’ enhanced seawater contents, reducing fracture-driving stresses by orders of magnitude and therefore greatly enhancing stability.

## Introduction

**Fig. 1:**
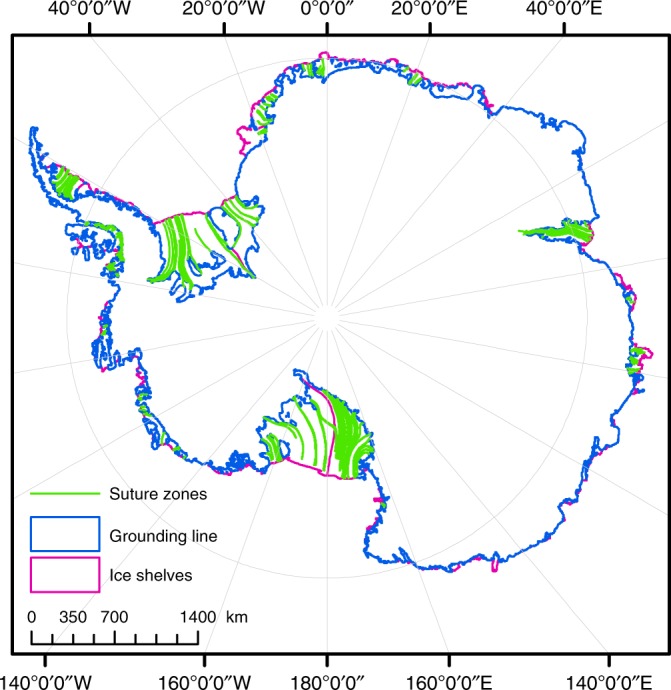
Principal suture zones on the Antarctic ice shelves. The shelves’ calving fronts and grounding lines are, respectively, shown in magenta and blue colours^52,60,61^. Suture zones are shown in green and are abundant on all major and most smaller ice shelves.

Half of the Antarctic coastline is fringed by ice shelves (Fig. 1) that are vulnerable to climate-driven retreat^1–4^. Around one quarter of the ice-shelf area has been lost on the Antarctic Peninsula in the past half century, and some shelves, including Larsen A and B, have largely disintegrated^4,5^. The final demise of the Larsen B Ice Shelf in 2002 triggered a twofold to fourfold acceleration of its former tributary glaciers that persists to the present day^6–8^, demonstrating the fundamental role that ice-shelf buttressing plays in regulating sea level rise. Current predictions of ice-shelf stability and future loss of grounded Antarctic ice are subject to considerable uncertainty, however, because the processes driving and resisting ice-shelf retreat are not well understood.

Antarctic ice shelves are commonly composed of meteoric ice and firn units derived from tributary glaciers and snow accumulation on the shelf, with units from adjacent tributaries fused together by suture zones^9,10^ (Fig. 1). Formed in the lee of peninsulas, ice rises or ice rumples protruding into the ice shelf, such zones are distinct in satellite images owing to smooth surfaces that bound relatively fractured meteoric ice, as typified by the Larsen C Ice Shelf on the Antarctic Peninsula (Fig. 1). By inhibiting the propagation of rifts and other fractures, suture zones thus appear to be critical for ice-shelf stability, although the physical properties and processes that allow them to do so are still unknown, as is the magnitude of stress modification they provide. Current ice-sheet models cannot therefore capture the stabilising influence of suture zones, so that simulations of ice-shelf retreat and grounded ice response may be subject to considerable uncertainty.

**Fig. 2:**
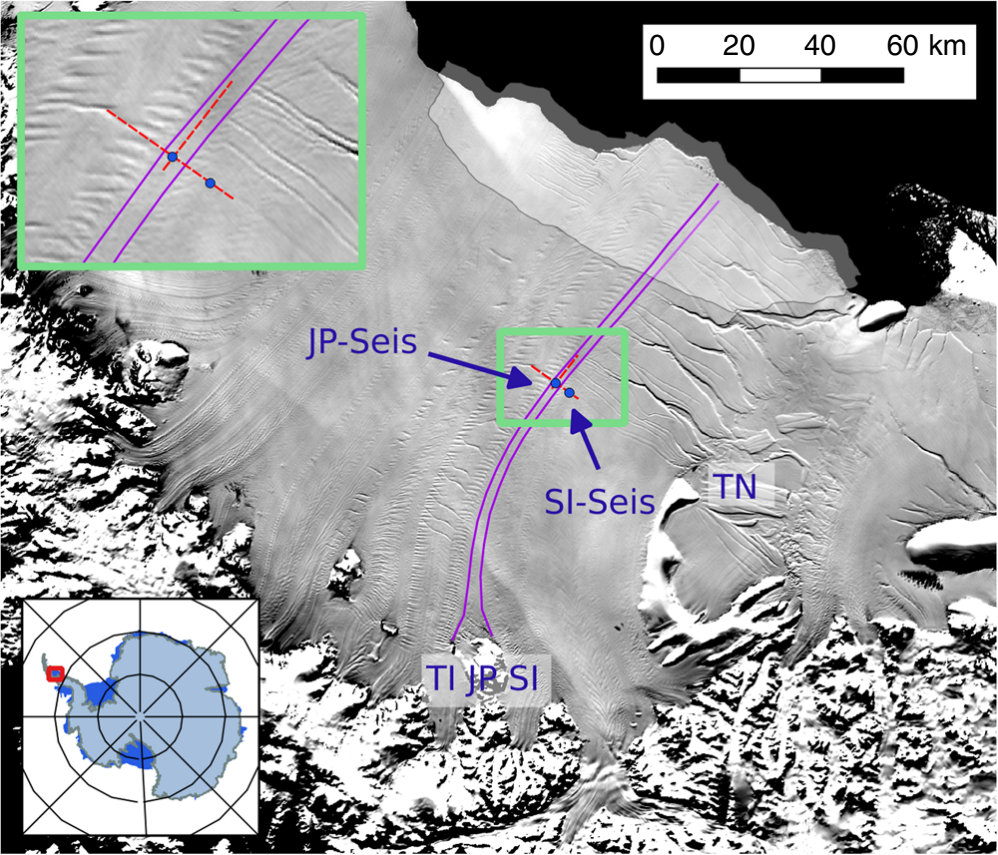
Larsen C study area in 2008/09. ASTER-GDEM [http://nsidc.org/data/docs/agdc/nsidc0516-cook/] derived DEM is superimposed on a 2008 MODIS image [https://earthdata.nasa.gov/data/near-real-time-data/rapid-response]. Inset shows location of Larsen C Ice Shelf on the Antarctic Peninsula, with Joerg Peninsula (JP), Trail Inlet (TI), Solberg Inlet (SI) and Table Nunatak (TN) labelled. The magenta lines trace the outlines of the JP suture zone, the first ~50 km of which is its formation area that is laterally compressed (white arrows) as TI- and SI-derived meteoric ice-shelf units converge. The filled blue circles mark the locations of our seismic reflection profiles at JP-Seis and SI-Seis, and the two red dotted arrows show our ground-penetrating radar (GPR) profiles across and along flow. The solid black arrow indicates the unnamed glacier on the JP that calves glacier-derived blocks into the JP suture zone, and the light grey shaded area indicates the large tabular iceberg (A68) that calved in July 2017^32,33^.

**Fig. 3:**
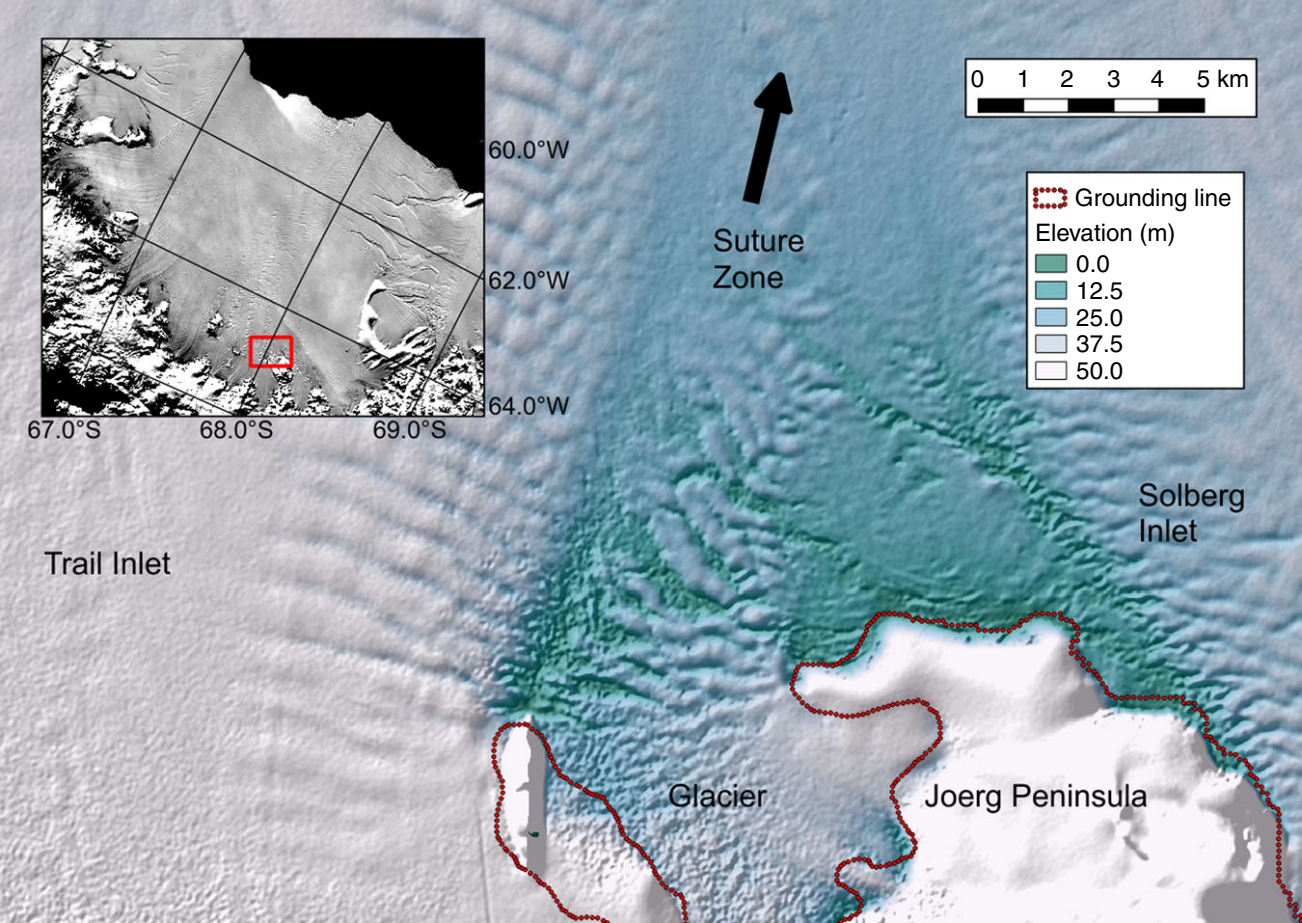
Formation area of the Joerg Peninsula (JP) suture zone. TanDEM-X Synthetic Aperture Radar (SAR)-derived interferometric DEM for 27 January 2012, as outlined by red box in inset. The red dotted line in the main image shows the grounding line. The unnamed glacier calves blocks of firn and meteoric ice into the formation area (ISU-3 in Fig. 4c, d), which are physically elevated (white colours) relative to the thinner mélange (ISU-4, green colours) that forms between them. This heterogeneous mixture of ice blocks and mélange is then compressed laterally into the nascent suture zone by the converging meteoric ice-shelf units derived from Trail and Solberg inlets (ISU-6 in Fig. 4c, d). The diminishing elevation differences between the glacier-derived blocks and the mélange with distance from the JP’s tip are then due to surface accumulation of the in situ firn (ISU-1) and meteoric ice (ISU-2) layers and the accretion of basal marine ice (ISU-5).

A suture zone in the south of the Larsen C Ice Shelf, formed in the lee of the Joerg Peninsula (JP) and thence embedded between meteoric ice units derived from feeding glaciers in the neighbouring Solberg and Trail Inlets (Figs. 2 and 3), is representative of these processes^11,12^. The cold and stiff ice of the tributary glaciers gradually converges downstream of JP (Fig. 2), leaving an opening in its immediate wake into which glacier-derived blocks calve from an unnamed glacier situated on JP (Figs. 2 and 3). The gaps between these ‘glacier-derived blocks’ (Fig. 3) are then filled by a mélange, the precise provenance of which is unclear^11,13^. We conjecture that the mélange may be composed of sea ice on top of which large amounts of snowfall and drifting snow have accumulated, owing to the formation zone forming a depression relative to the bounding ice-shelf surface (Fig. 3). Smaller icebergs calved from the peninsula may hypothetically also add to this mix. In such a situation, the surface snow loading would force the nascent melange downwards, allowing it to be flooded by seawater, which could also flood laterally into the firn portion of the glacier-derived blocks either before or after calving^14–18^. Finally, marine ice may accrete at the base of the nascent suture zone as seawater rises from the sub-shelf cavity into basal hollows, where it would super-cool and freeze^11,14^. According to our conjecture, lateral convergence of the tributary glaciers may then compress the mélange and the glacier-derived blocks into the emerging suture zone^11,13^.

Both the mélange and the accreted basal ice are largely of marine origin and therefore have a temperature that is much closer to seawater (~−0.5 °C to −2 °C)^19–22^ than the relatively cold meteoric ice and firn derived from the ice-sheet interior or accumulated on the shelf (~−5 to −15 °C on the Larsen C Ice Shelf)^23^. Suture zones are therefore expected to be warmer, softer, more deformable and more heterogeneous in provenance than the cold meteoric ice units that enclose them^10–14,31^. Although actual in situ measurements of these anomalous characteristics of suture zones are very sparse, they are hypothesised to cause stress relief ahead of incipient fractures, thereby detaining them and resulting in the observed, less fractured suture-zone surfaces^10–14,24–34^. The JP suture zone, for example, has detained multiple rift sequences that originate at Table Nunatak, some 100 km downstream of JP (Fig. 2), so that the Larsen C Ice Shelf would be much smaller without this suture zone^33^. Our study therefore focusses on a critical component of the Antarctic Ice Sheet.

Here we address the current paucity of in situ observations by presenting new integrated field data of the JP suture zone. These data allow us to evaluate the widely held hypothesis that warmer temperature^10–14,31^ and associated ice softening and enhanced deformation are the root causes of fracture detainment.

## Results

### Structure of the JP suture zone

**Fig. 4:**
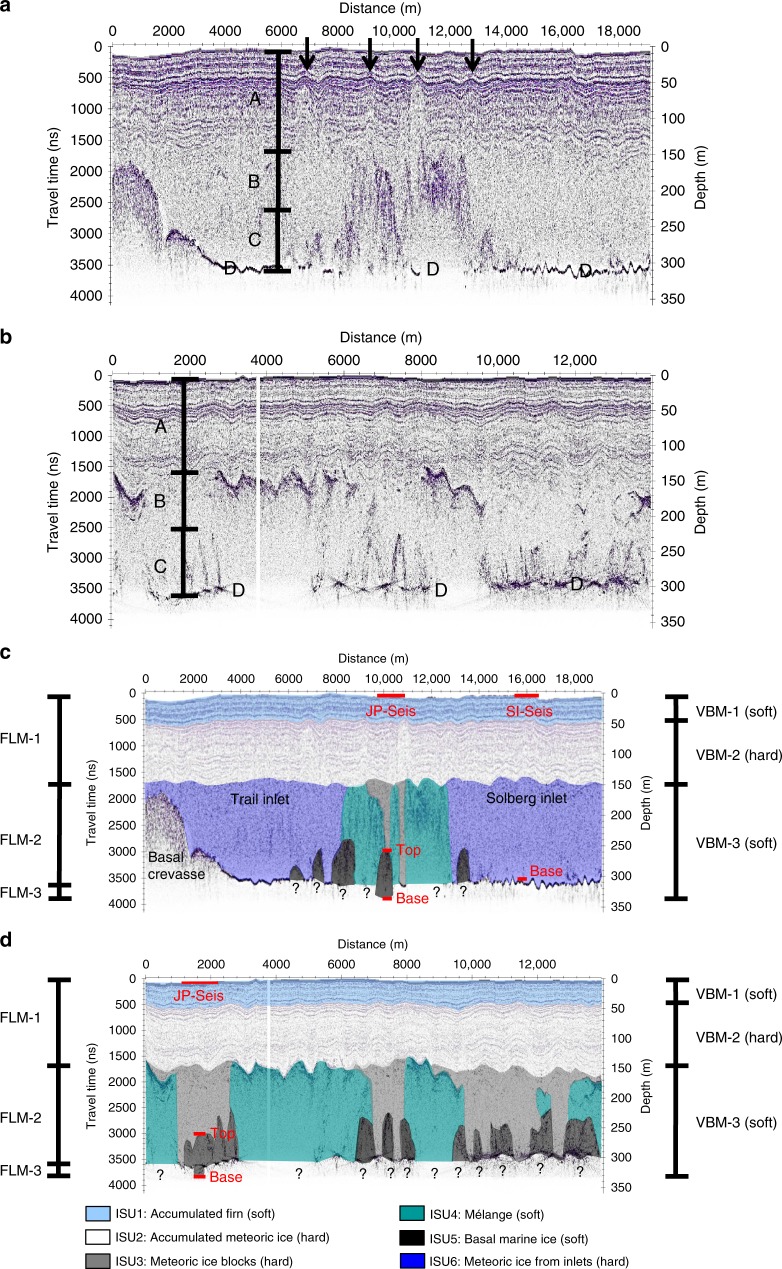
Ground-penetrating radar (GPR) profiles of the Joerg Peninsula (JP) suture zone. See Fig. 2 for profile locations and directions. **a** Profile across the suture zone. **b** Profile along the suture zone. In both **a** and **b**, the main observations are labelled, including (Type A) layered horizontal reflections from the ice-shelf surface to depths of ~140 m that exhibit buckling within the suture zone, where four prominent buckle stacks are indicated by the four black arrows in **a**; (Type B) events between depths of ~140 and ~220 m that appear as scattered hyperbolae or locally continuous reflections, respectively, in **a** and **b**; (Type C) events deeper than ~220 m that have distorted or disrupted hyperbolic shapes; and (Type D) reflections from the ice-shelf base. **c** Interpreted profile across the suture zone. **d** Interpreted profile along the suture zone. The solid red lines in **c** and **d** mark the total seismic profile lengths at JP-Seis and SI-Seis at the ice surface and at depth also mark the top of the basal marine ice (‘Top’) and the ice-shelf base (‘Base’), as delineated, respectively, by our GPR and seismic data. The depths of the bases of the mélange and basal marine ice units are unknown, as indicated by the black question marks. In both **c** and **d**, the three main layers considered by our flowline model (FLM) are labelled, including a surface layer (FLM-1) integrating accumulated firn (ISU-1) and meteoric ice (ISU-2), a central layer (FLM-2) integrating advected glacier ice (ISU-3) and mélange (ISU-4) and a basal layer of accreted marine ice (FLM-3, equivalent to the ISU-5) (Fig. 5). Also labelled in both **c** and **d** are the three main layers considered by our viscous buckling model (VBM), including two soft layers—firn (VBM1, equivalent to ISU-1) and the conglomerate layer (VBM3) of mélange (ISU-4), basal marine ice (ISU-5) and meteoric ice blocks (ISU-3)—that enclose the hard layer of in situ meteoric ice (VBM2, equivalent to ISU-2).

We conducted ground-penetrating radar (GPR) and multi-component seismic reflection surveys to the north of Table Nunatak in December 2008/09, one centrally on the JP suture zone (JP-Seis) and one ~5 km to the south of it on the meteoric ice flow unit derived from Solberg Inlet (SI-Seis) (Fig. 2). Of the two representative GPR profiles shown here, the first runs from north (Trail-Inlet derived meteoric ice-shelf unit (ISU)) to south (Solberg-Inlet derived meteoric ISU) through location SI-Seis, crossing the JP suture zone through location JP-Seis. The second profile runs along the JP suture zone from west to east and intersects the cross-profile at JP-Seis (Fig. 2). As labelled in Fig. 4a, b, our GPR data are characterised by layered horizontal reflections from the ice-shelf surface to depths of ~140 m that exhibit buckling within the JP suture zone (Type A); events between depths of ~140 and ~220 m that appear as scattered hyperbolae or locally continuous reflections respectively in across (Fig. 4a) and along (Fig. 4b) flow profiles (Type B); events deeper than ~220 m that have distorted or disrupted hyperbolic shapes (Type C); and reflections from the base of the ice shelf that dominate in the meteoric ice units derived from Solberg and Trail inlets and appear also in selected areas of the JP suture zone where type B and C events are absent (Type D). The layered horizontal reflections of type A are laterally continuous both across (Fig. 4a) and along (Fig. 4b) the suture zone. Each continuous reflection represents a former ice-shelf surface in the suture-zone’s formation area, which is then buried downflow by ongoing surface accumulation of ‘in situ’ firn and compaction to meteoric ice^13,35^. Of the 140 m thickness, around 40–45 m are in situ firn^29^ and approximately the lower 100 m are in situ meteoric ice. The provenance of these in situ firn and in situ meteoric ice layers is therefore distinct from the underlying glacier-derived blocks.

Observed previously in our GPR data^11–13^, type B events are typically discontinuous and characterised by overlapping discrete hyperbolae, and coherent energy returns from below them are normally absent (Fig. 4a, b). These events are therefore compatible with GPR energy being diffracted within the upper portions of mélange, with the extinction of the underlying signal caused by its strongly elevated electrical conductivity^11–15,36^ (Fig. 4c, d).

In contrast, the areas marked by gaps between type B events are consistent with the presence of glacier-derived blocks (Fig. 4c, d). Coherent energy returns are sometimes visible beneath marginal portions of type B events (Fig. 4a, b) and are likely caused by reflections from the sides of the profile line or possibly some localised flooding of snow and firn located on top of the glacier-derived blocks. Our seismic surveys at JP-Seis were recorded above an ice block (Fig. 4c, d), which is 100 m thick at this location with an event of type C marking its base (Fig. 4a, b).

Events of type C are more diffuse than those of type B, especially in the along-flow direction, and visually resemble the shapes of basal crevasses (Fig. 4a, b). However, in this case our observations are not consistent with the presence of open basal crevasses because type C reflections at depth are not commonly accompanied by surface depressions, as would be expected owing to hydrostatic adjustment of the shelf^37,38^. We furthermore note that GPR signal returns are typically absent from beneath type C events just as they are from beneath type B events (Fig. 4a, b), and the strongly elevated electrical conductivity of basal marine ice together with increased energy scattering are again the most likely explanations for this observation. In the absence of any other plausible explanation, we therefore attribute the type C events to the presence of basal marine ice that has accumulated in basal crevasses and other concave undulations at the base of the suture zone. This inference is supported by analysis of our seismic data, outlined below.

In summary, our GPR data and associated observations are consistent with the presence of six main ISUs composing the JP suture zone at JP-Seis and the adjacent inlet-derived ice (Fig. 4c, d). These include (ISU-1) ~40–45-m-thick in situ firn that overlies (ISU-2) ~100-m-thick in situ meteoric ice, which in turn overlies (ISU-3) glacier-derived blocks that alternate laterally with (ISU-4) an ice mélange likely composed of amalgamated sea ice and (ISU-5) basal marine ice. The JP suture zone is (ISU-6) surrounded by meteoric ice derived from the Trail and Solberg inlets (Fig. 4c).

### Along-flow evolution of the JP suture zone

**Fig. 5:**
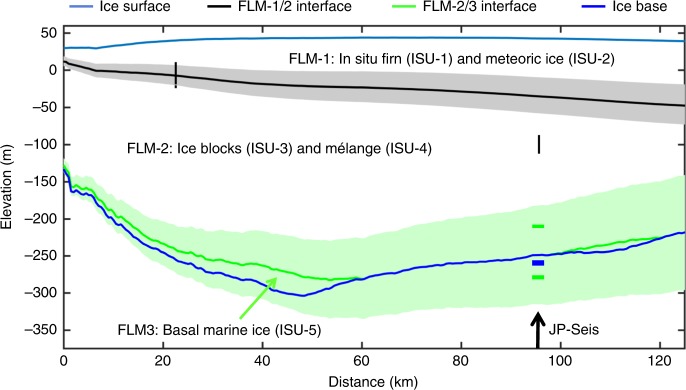
Modelled flowline along the JP suture zone. The tip of the JP is located at distance 0 km and JP-Seis is at 95 km, as labelled. The continuous horizontal blue lines represent the ice-shelf surface and base. The continuous horizontal black line represents the simulated interface between the in situ firn (ISU-1) and meteoric ice (ISU-2) layers accumulated on the shelf (layer FLM-1 in our flowline model) and the upper surfaces of the glacier-derived ice blocks (ISU-3) and mélange (ISU-4) (layer FLM-2). The horizontal green line represents the simulated ‘marine ice interface’ between these ice blocks/mélange and the basal marine ice (layer FLM-5, equivalent to ISU-5). The shaded bounds represent the cumulative sensitivity of the model to the input parameters (see ‘Methods’). The short vertical black lines represent depth estimates of the in situ meteoric interface from our GPR data, where their lengths indicate the inherent uncertainties. The short horizontal blue and green lines at JP-Seis indicate, respectively, the ice shelf base and the top and base of the basal marine ice body delineated by our seismic surveys, where line thicknesses indicate the inherent uncertainties.

To place the section we have imaged with GPR into a broader spatial context, we use our established one-dimensional (1-D) flowline model^13,20^, initiated at the JP’s grounding line. We simulate the along-flow evolution of a surface layer of in situ firn and meteoric ice (FLM-1 in Fig. 4c, d, integrating ISU-1 and ISU-2), a central layer of ice with initial thickness equal to that observed at the grounding line (FLM-2 in Fig. 4c, d, integrating ISU-3 and ISU-4), and a layer of marine ice accreted to the base of FLM-2 (FLM-3 in Fig. 4c, d). We emphasise that our flowline model is only able to simulate marine ice accretion to the base of FLM-2 but cannot explicitly account for any such accretion into basal crevasses and other concave undulations at the base of the suture zone. The initial thicknesses of the surface and basal layers are assumed to be zero at the grounding line, and the central ice layer is treated as a continuum irrespective of its provenance as either ISU-3 or ISU-4. We test the cumulative sensitivity^13^ of the simulated surface and basal layers to changes of ±20% in along-flow velocity, surface mass flux rate and vertical strain rate and ±0.4 m yr^−1^ in basal mass flux rate (shaded bounds in Fig. 5). Focussing on the suture-zone central flowline^10,29^ that traces through JP-Seis (Fig. 2), the model accumulates a surface layer ~65 ± 20 m thick and a basal layer between 0 and ~75 m thick halfway (47.5 km) between the JP’s tip (0 km) and location JP-Seis (95 km; Fig. 5). The thickness of the modelled surface layer steadily increases to reach ~80 ± 25 m at JP-Seis, where the basal layer is between 0 m and ~65 m thick. The presence and thickness of marine ice is particularly sensitive to input parameters, including the modelled basal melt and accretion rate.

The modelled interface between the surface layer and the central layer agrees closely with previous GPR measurements ~22 km downflow of the JP’s tip^11^ but underestimates the depth of this interface by ~50 ± 20 m at JP-Seis (Fig. 5). This may be at least partially explained by the considerable variability in interface depth over short distances in the across-flow direction or by changes in surface accumulation or ice flow velocity during the ~300-year timescale of advection between the JP’s tip and JP-Seis (Fig. 4a, c). The bounds of our modelled basal marine ice layer (Fig. 5) enclose the discrete bodies of basally accreted marine ice inferred from our GPR data (black shaded bodies in Fig. 4d). Next, we evaluate and refine the GPR-based interpretations through analysis of independent seismic data.

### Seismic analysis of elastic ice properties at SI-Seis

**Fig. 6:**
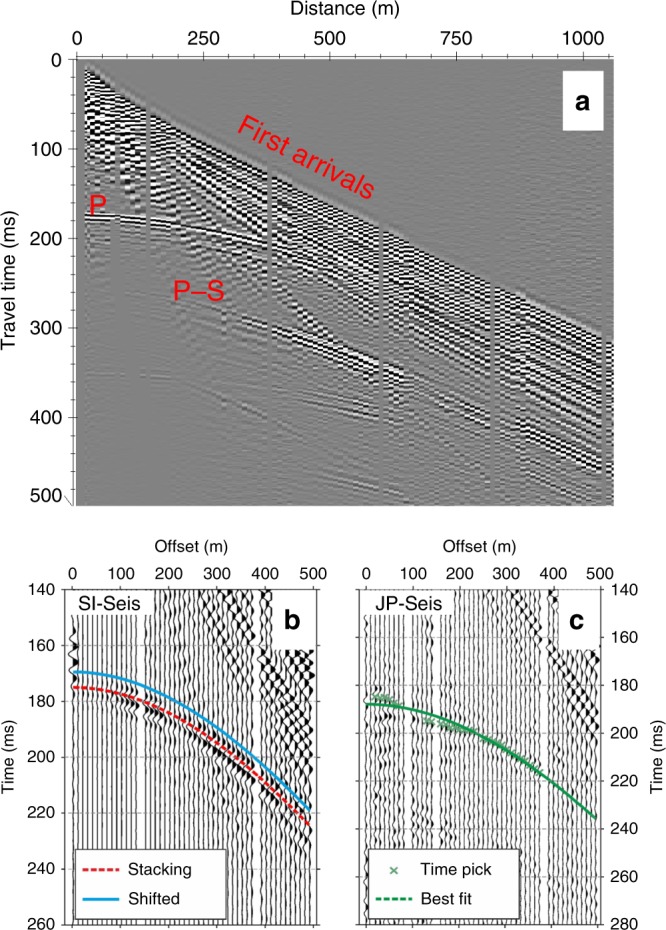
Interpretation of seismic reflections from the ice-shelf base at SI-Seis and JP-Seis. **a** Walk-away seismic reflection data showing first arrivals, P-wave reflection and P-S conversion from the ice-shelf base at SI-Seis, as used in our analyses. **b** Interpreted P-wave reflection from ice-shelf base at SI-Seis. **c** Interpreted P-wave reflection from ice-shelf base at JP-Seis. The base ice reflections at SI-Seis are readily identified and back shifted (see ‘Methods’), while those at JP-Seis are not and therefore represented by a best-fit hyperbola.

**Fig. 7:**
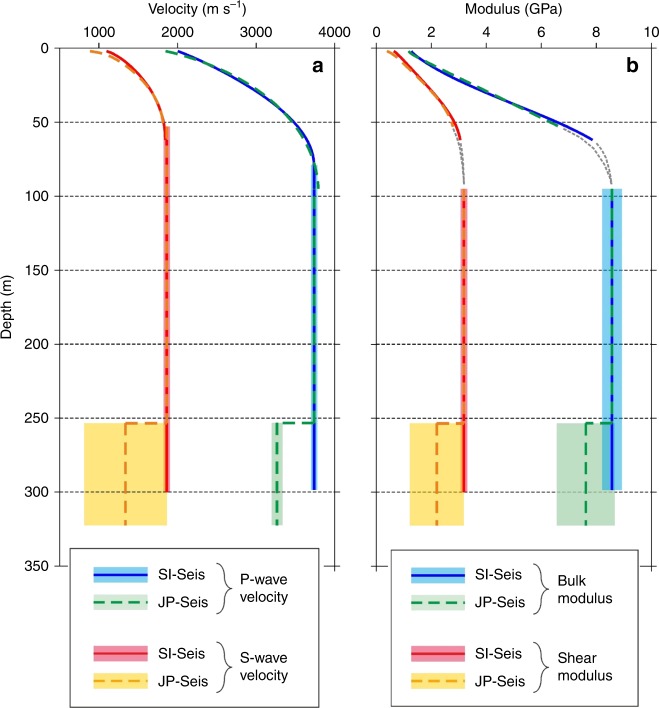
Depth profiles of seismic and elastic attributes at SI-Seis and JP-Seis. **a** P-wave and S-wave velocities. **b** Bulk and shear moduli. In both **a** and **b**, the shaded bounds represent possible ranges of seismic velocity models derived from Monte Carlo analysis, considering the median plus the interquartile range (see ‘Methods’).

Our seismic data from SI-Seis on the meteoric ISU to the south of the JP suture zone (Fig. 2) are characterised by first arrivals refracted through firn layers, P-wave reflections from the ice-shelf base and a strong P-S mode conversion (Fig. 6a). Refraction and normal moveout analyses of these phases (see ‘Methods’) yielded seismic velocity profiles through the firn and ice column, where the P- and S-wave velocities of meteoric ice were estimated to be 3739 ± 41 and 1864 ± 18 m s^−1^, respectively (Fig. 7a). Depth conversion of these travel times then indicates an ice-shelf thickness of 298 ± 2.5 m at SI-Seis (Fig. 7a), in close agreement with our GPR data (Fig. 4). If the density of the ice fraction is 917 kg m^−3^, Hashin–Shtrikman (H-S) bounds and the Voigt–Reuss–Hill (VRH) average (see ‘Methods’) suggest maximum bulk and shear moduli in meteoric ice of 8.57 ± 0.36 and 3.19 ± 0.12 GPa, respectively (Fig. 7b).

### Seismic analysis of elastic ice properties at JP-Seis

Our seismic data at JP-Seis were recorded above a glacier-derived block, the base of which is marked by a reflection event of type C at a GPR-derived depth of 253.5 m (Fig. 4). Of this thickness ~40–45 m are in situ firn^29^, and the remaining ~210 m are consistent with in situ meteoric ice overlying the meteoric ice of the glacier-derived block (Fig. 4). The depth profiles of P- and S-wave velocities through the firn at JP-Seis (Fig. 7a), and by inference also the bulk and shear moduli (Fig. 7b), are indistinguishable from those at SI-Seis. We therefore assume that the bulk P- and S-wave velocities and bulk and shear moduli of the meteoric ice inferred at SI-Seis also apply to the meteoric ice block at JP-Seis. The bulk P-wave velocity of the ice of marine origin below the type C event at JP-Seis is then estimated to be 3264 ± 63 m s^−1^, 13% lower than that of the meteoric ice in the glacier-derived block (3739 ± 41 m s^−1^; Fig. 7a). The ice-shelf thickness at JP-Seis is then predicted to be 322.5 ± 1.5 m, with a 69-m-thick body of basal marine ice beneath the type C event (Fig. 7). The ice shelf therefore appears to be about 20 m thicker at JP Seis (322.5 ± 1.5 m) than at SI-Seis (298 ± 2.5 m) owing to the presence of basal marine ice.

The ~13% reduction in bulk P-wave velocities (from 3739 ± 41 to 3264 ± 63 m s^−1^) from the meteoric ice in the glacier-derived block to the basal marine ice cannot be explained by a change in ice density or temperature alone. Because seismic velocities are inversely proportional to the square root of ice density, this velocity reduction would require an implausible increase in ice density (to ~1200 kg m^−3^). Because seismic velocities are also only weakly dependent on ice temperature^39^, a change of >40 °C is required to change P-wave velocity by 100 m s^−1^—a temperature change also cannot explain the measured decrease in bulk P-wave velocity change of ~475 m s^−1^. Because P-wave velocities are much lower in seawater (~1500 m s^−1^) than in ice (~3200–3700 m s^−1^, above), the proposed presence of liquid seawater in the pore space of basal marine ice offers the most plausible physical means of explaining the large observed P-wave velocity reduction^40,41^.

**Fig. 8:**
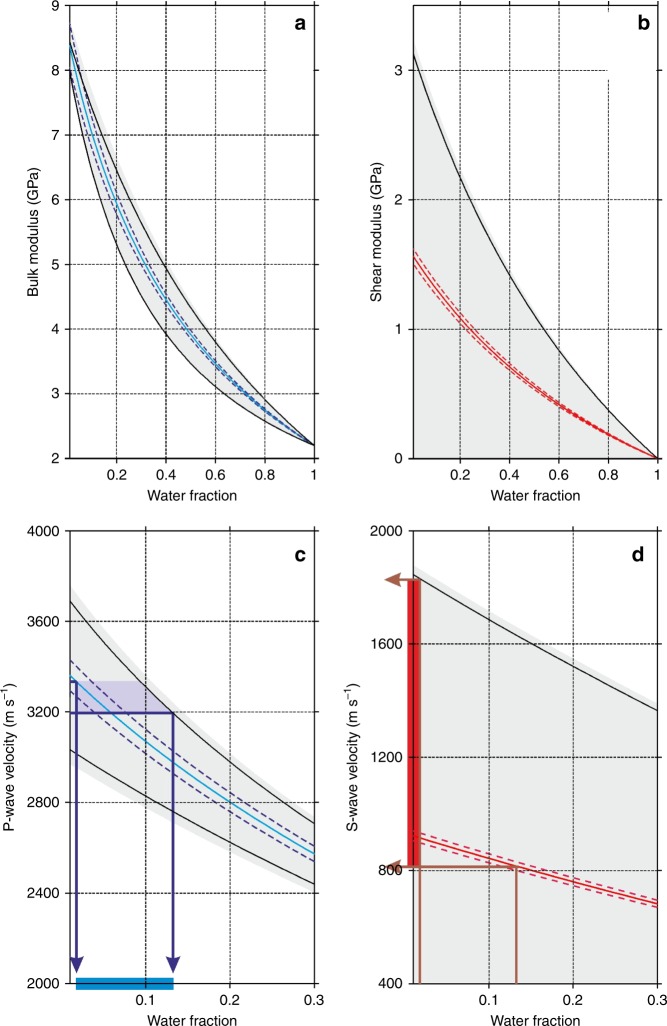
Elastic parameters of basal marine ice (ISU-5) derived from the Hashin–Shtrikman (H-S) bounds. The analysis assumes the presence of a mixture of ice and seawater of varying fractions. Grey areas show the full range of the H-S bounds in dependence of seawater fraction, for **a** bulk modulus ($K_{{\mathrm{HS}}}^ +$ in Eq. (2)); **b** shear modulus ($\mu _{{\mathrm{HS}}}^ +$ in Eq. (3)); **c** P-wave velocity; and **d** S-wave velocity (see ‘Methods’). Solid blue and red lines show the Voigt–Reuss–Hill averages, above which the H-S bounds describe a matrix-supported porous solid. In **c**, the observed P-wave velocity range of basal marine ice (3264 ± 63 m s^−1^) implies a seawater fraction of ~2–13%. In **d**, this seawater fraction range then implies an S-wave velocity range of basal marine ice of 813–1853 m s^−1^. All velocity calculations assume a density of 915 kg m^−3^ for meteoric ice and 920–990 kg m^−3^ for basal marine ice.

To estimate the seawater content of the basal marine ice at JP-Seis, we define H-S bounds of bulk and shear moduli (Fig. 8a, b), and by inference of bulk P- and S-wave velocities (Fig. 8c, d), as a function of seawater fraction. In doing so, we assume that the seawater contents of the overlying firn and meteoric ices are negligible and that seawater has a fixed density of 1020 kg m^−3^. By using the VRH average, the bulk P-wave velocity of 3264 ± 63 m s^−1^ measured for the basal marine ice at JP-Seis then implies a seawater content of 2–13% (Fig. 8c) and an S-wave velocity between 813 and 1853 m s^−1^ (Figs. 7a and 8d). The bulk and shear moduli of the basal marine ice are then estimated to be 8.36 ± 0.42 and 1.88 ± 1.27 GPa, respectively (Fig. 7b).

### Analysis of viscous ice properties

Although our seismic measurements provide unique observational evidence of meteoric and basal marine ice properties, including the latter’s pore seawater contents, they have two main limitations. First, they impose short-term transient strains and thus sample the elastic properties of ice, while, in reality, ice is considered to deform by secondary creep in response to long-term strain. The inferred elastic properties can therefore only serve as an approximation of the actual physical properties that control the deformation of ice. Second, our seismic experiments sampled a glacier-derived block calved from the JP but not the mélange that fuses these blocks together (Fig. 4). To complement our seismic analyses and overcome these limitations, we characterise the suture-zone’s long-term strain response by analysing folds in the ice apparent from our GPR data, using viscous buckling modelling (VBM) commonly applied to geological strata^42^ (see ‘Methods’).

Undulating reflections are common in our GPR data, but they are particularly pronounced at depths of ~40–140 m in the JP suture zone (Fig. 4a). This depth range corresponds to hard in situ meteoric ice (layer VBM-2, equivalent to ISU-2), with ~40 m of soft firn above (VBM-1, equivalent to ISU-1) and ~110 m of layer VBM-3. Layer VBM-3 integrates ISU-3, ISU-4 and ISU-5 into a continuum and is therefore softer, on average, than the harder meteoric ice layer VBM-2 above. It thus appears that the harder layer VBM-2 is sandwiched between two softer shelf units above (VBM-1) and below (VBM-3) (Fig. 4c, d). This situation typifies the conditions necessary for viscous buckling of the hard in situ meteoric ice (VBM-2) to occur, which we attribute to lateral compression of the JP suture zone between the converging Solberg and Trail inlets (Figs. 2 and 3). The softer units above (VBM-1) and below (VBM-3) appear less buckled because they can deform more easily. Furthermore, less lateral compression will have occurred with distance from the JP’s formation area as convergence rate decreases (Fig. 2), so that firn accumulated later on the shelf will also appear less buckled (Fig. 4a, b).

At least four diagnostic buckle stacks are observed in our GPR cross-section (marked by black arrows in Fig. 4a), where the harder in situ meteoric ice is up to *h* ≈ 100 m thick with characteristic wavelengths of up to *λ* ≈ 1000 m (Fig. 4a). Allowing for reasonable ranges of *h* (50 ≤ *h* ≤ 100 m) and *λ* (500 ≤ λ ≤ 1000 m) and using an appropriate rate factor range for this meteoric ice (2.9 × 10^−25^ ≤ *A*_h_ ≤ 1.6 × 10^−24^ s^−1^ Pa^−3^ for –15 ≤ *T* ≤ –5 °C; see ‘Methods’), the combined rate factor of the combined softer units of firn, mélange and basal marine ice has a 90% confidence interval of ~1 × 10^−24^ to ~5 × 10^−22^ s^−1^ Pa^−3^ (Fig. 8a) with an equivalent temperature (*T*) range of ~−5 °C to +17 °C (Fig. 8b). Only a maximum of 15% of samples therefore lie within a realistic temperature range of *T* < 0 °C. From a statistical perspective, temperature enhancement is therefore unlikely to be the sole cause of suture-zone softening and its ability to detain fractures.

This apparent paradox can readily be reconciled if we allow for the presence of seawater in the pore space of the basal marine ice and mélange. The seawater fraction equivalent to the combined rate factor range of the combined softer units of firn, mélange and basal marine ice (*A*_s_, ~1 × 10^−24^ ≤ *A*_s_ ≤ 5 × 10^−22^ s^−1^ Pa^−3^) is ~2–12% (Fig. 8c). The 90% confidence interval for the rate factor ratio between the harder (*A*_h_, in situ meteoric ice) and the combined softer (*A*_s_) units is therefore ~6–100 so that, for a given stress, the combined softer units will deform between one and two orders of magnitude more readily than the harder in situ meteoric ice. Conversely, the ratio in the flow law parameter ${\mathrm{B}} = \root {3} \of {{\mathrm{A}}}$ is 1.8–4.6, expressing the excess stress required to produce a given strain rate in the in situ meteoric ice relative to the combined softer units of firn, mélange and basal marine ice.

## Discussion

GPR and seismic measurements revealed the JP suture zone in the Larsen C Ice Shelf to be heterogeneous both vertically and laterally at the local scale and along flow and across flow at the ice-shelf scale. The suture zone is composed of five main units (Fig. 4), including in situ firn (ISU-1), in situ meteoric ice (ISU-2), glacier-derived blocks calved from an unnamed glacier on the JP (ISU-3, Figs. 2 and 3), mélange of unconfirmed provenance that fuses these blocks together (ISU-4) and basal marine ice accreted from below (ISU-5). The shelf ice on either side of the JP suture zone is derived from the Train and Solberg inlets, forming ISU-6. Our seismic surveys were positioned above a glacier-derived block (ISU-3, Fig. 4c, d) and allowed us to estimate the thicknesses and physical properties of ISU-1, ISU2 and ISU-3 combined and ISU-5 (Figs. 4, 7 and 8). The bulk seismic velocities (Fig. 7a) and elastic moduli (Fig. 7b) of basal marine ice were notably lower than those of the meteoric ice above, an observation that cannot be explained by ice density changes or temperature enhancement alone. We have shown instead that a combined seawater content of mélange (ISU-4) and basal marine ice (ISU-5) of 2–13% can explain these bulk velocity and moduli reductions (Fig. 8).

**Fig. 9:**
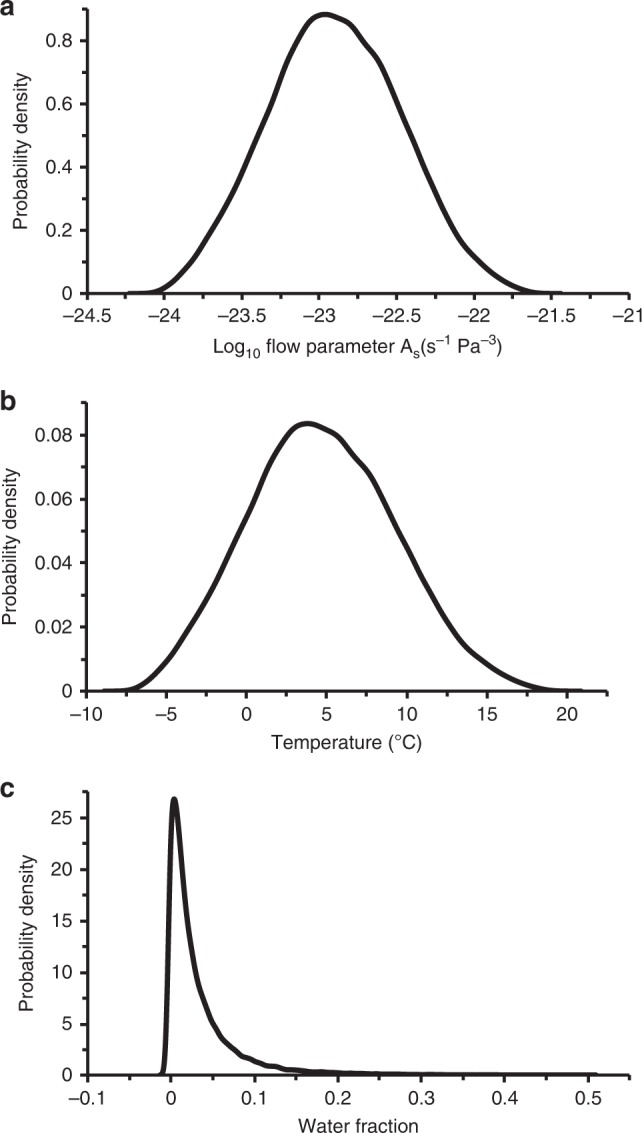
Viscous buckling analysis of GPR data. Estimated probability densities of bulk suture-zone properties are shown, including **a** combined rate factor (*A*_s_ in Eq. (4)) for the softer layers VBM-1 (equivalent to ISU-1) and VBM-3 (integrating ISU-3 to ISU-5) (Fig. 4c, d), **b** ice temperature (*T* in Eq. (5)) for these layers and **c** ice seawater fraction for these layers (*W* in Eq. (6)).

Complementary analysis of folds revealed by our GPR data (Fig. 4a) using established geological theory of viscous buckling captured the suture-zone’s long-term bulk deformation. This deformation particularly reflects lateral compression in the zone’s formation area (Figs. 2 and 3), buckling the harder unit of in situ meteoric ice (ISU-2) while the combined softer units of firn (ISU-1), mélange (ISU-4) and basal marine ice (ISU-5) appear less buckled owing to their greater ability to deform (Fig. 4a). The geometry of the buckles in the harder in situ meteoric ice (ISU-2) were used to diagnose the contrast in physical properties between it and the combined softer units ISU-1 and ISU-3 to ISU-5 (Fig. 9). We found, again, that temperature enhancement cannot be the sole cause of these units’ anomalous softness (Fig. 9a, b), while a seawater content of 2–12% can (Fig. 9c).

Because firn (ISU-1) only contributes ~40 m (~20%) to the total thickness of the combined softer units, these inferences particularly apply to mélange (~110 m or ~50%) and basal marine ice (~70 m or ~30%) (Figs. 4, 5 and 7) that dominate the combined softer units’ anomalously large strain rate response to the imposed stress. We infer that mélange (ISU-4) and basal marine ice (ISU-5) have substantially enhanced seawater contents (Figs. 8c and 9d) and will therefore deform between one and two orders of magnitude more readily in response to the same stress than the harder in situ meteoric ice (ISU-2).

Both our seismic and viscous buckling analyses imply seawater contents between ~2% and ~12–13% (Figs. 8c and 9d). There is an important distinction in that the seismic analysis applies only to the basal marine ice (ISU-5), while viscous buckling calculations apply collectively to it and the mélange (ISU-4). The two approaches also sample different portions of the JP suture zone. Basal marine ice accretion and lateral compression are both strongly focussed within the zone’s formation area extending ~50 km off the tip of the JP (Fig. 2)^11^, a distance over which, according to our flowline model (Fig. 5), more than ~80% of the in situ firn and meteoric ice accumulates. It is therefore likely that both buckle formation and the evolution of mélange and basal marine ice by simultaneous accretion, lateral compression and burial are also concentrated within this zone. Our viscous buckling calculations therefore sample the properties of mélange and basal marine ice in the suture-zone’s formation area, even though the buckles were observed at JP-Seis. Together these calculations and our seismic inferences therefore imply that the average seawater content of mélange and basal marine ice in the suture-zone’s formation area is of a similar range (i.e., ~2% to ~12–13%) to that of basal marine ice at JP-Seis.

The accretion rate of marine ice to the base of ice shelves^20,43–45^ or in rifts or basal crevasses^21,46^, and therefore the physical and chemical properties of marine ice, can vary widely depending on the ocean temperature and the glaciological setting. The compaction of the basal marine ice layer of the Amery Ice Shelf, Antarctica increases upwards away from the ocean interface^20^. Similarly, ice forming the base of a rift in King Baudouin Ice Shelf, Antarctica shows a gradual decrease in compaction over some tens of metres, eventually disaggregating into unconsolidated platelets^47^. This progression was divided into three material facies, with basally accreted platelets underlying older marine ice (formed by the consolidation and drainage of those platelets), underlying granular ice (formed by surface-derived snow or firn being invaded by seawater). In both the Amery and the King Baudouin ice shelves, therefore, thick layers of marine ice that had accumulated over extended periods of time exhibit either internal gradation or discrete layering. Two types of seismic evidence support the hypothesis that basal marine ice beneath the Larsen C Ice Shelf may be similarly layered. First, our own (compare Fig. 6b, c) as well as previously published data^48^ are characterised by poorly defined primary P-wave reflections from the base of suture zones, while this reflection commonly is strong elsewhere. Second, while the interface between the glacier-derived block of meteoric ice and the basal marine ice is sharply defined in our GPR data (Fig. 4a, b), we do not observe an equally distinct seismic reflection from this interface (Fig. 6c). These two observations are best explained by gradational basal marine ice (ISU-5, Fig. 4c, d) whose upper and lower portions have relatively reduced and increased porosities and seawater contents, respectively, so that the corresponding acoustic impedances differ sharply from that of both the overlying meteoric ice block and the seawater in the underlying ocean cavity.

Experimental work previously found pronounced softening of ice when its seawater content increases from ~0.008% to ~0.8%^49^. Irrespective of the actual physical properties of mélange and basal marine ice, we can therefore assert that even our lowest inferred value of 2% seawater content would result in strong softening compared to the in situ meteoric ice (Figs. 8c and 9c). The combined thickness of softer mélange (ISU-4, ~110 m), basal marine ice (ISU-5, ~70 m) and firn (ISU-1, ~40 m) is ~220 m, which is substantially larger than that (~100 m) of the harder unit of in situ meteoric ice (ISU-2, Fig. 4c, d). Softer units thus constitute ~70% of the total suture-zone thickness (~320 m) in these areas. Even where glacier-derived blocks of meteoric ice (ISU-3) are present instead of mélange (Fig. 4c, d), the combined thickness of softer basal marine ice (~70 m) and softer firn (~40 m) is still ~110 m or ~35% of total suture-zone thickness (~320 m). In either case, the depth-averaged softness of the suture zone will therefore be noticeably greater than the surrounding ISUs derived from feeder glaciers in the Trail and Solberg Inlets (Figs. 2 and 3).

There are two main mechanisms whereby suture zones could detain fractures, as exemplified by the rifts that meet the JP suture zone after travelling northwards from Table Nunatak through the cold meteoric ice from Solberg Inlet (Fig. 2). First, suture-zone mélange (ISU-4) and nascent basal marine ice (ISU-5) are structurally and mechanically heterogeneous and will therefore be particularly susceptible to micro-crack formation ahead of the rift tip, as proposed previously for a large tabular iceberg calving event on the Amery Ice Shelf^50^. Micro-crack formation will reduce the stress intensity around the rift tip^50^ and could therefore cause rifts to be detained by suture zones, at least on a transitory basis. Only once a critical micro-crack density is reached can these micro-cracks coalesce to propagate the main rift^50^. Second, stress intensities around the rift tip could be reduced by viscous relaxation of softer suture-zone ice relative to meteoric ice that is colder and contains less liquid seawater. This would again blunt the rift tip and thus make it less likely that the rift would propagate. Either way, a rift will be more likely to break through a suture zone if it intersects an area dominated by harder glacier-derived meteoric ice than softer mélange (Figs. 2 and 4d). Indeed, the regional stress field will likely determine to what degree an incident rift will preferentially be diverted towards mélange or towards a glacier-derived meteoric ice block, a process that may demand further investigation.

The spatial arrangement and precise geometry of glacier-derived blocks (ISU-3) and units of mélange (ISU-4) and basal marine ice (ISU-5) will then further modulate the propagation of the rift tip in space and time. For example, in a cross-section through the suture zone at JP-Seis the combined mélange and basal marine ice units are wider near the ice-shelf base than higher up in the ice column (Fig. 4a). A full-thickness rift propagating northward from Table Nunatak and eventually meeting the JP suture zone at right angles would therefore encounter basal marine ice first, especially if the regional stress intensity is such that the rift is propagating fastest at the base. In contrast, the part of the rift above the basal marine ice (top 75–80% of suture-zone thickness, Fig. 4a) would continue to propagate through the meteoric ice from the Solberg Inlet until eventually encountering mélange. An even more complex situation would emerge if the liquid seawater content varied spatially within basal marine ice and mélange. In this regard, the heterogeneity of the suture-zone’s anatomy, both vertically and laterally across and along the suture zone, may well control the detailed processes of rift propagation and likelihood of rifts breaking through it, with potential implications for ice-shelf stability.

The fact that suture-zone heterogeneity may play a major role in ice-shelf stability is daunting from the perspective of predictive ice-shelf modelling, because this requires detailed knowledge of englacial structures and physical properties. Alleviating this concern, we have (i) shown that simple analysis of viscous buckles apparent in cross-flow radargrams of suture zones can quantify their modulation of long-term strain rates; (ii) deciphered the signatures of glacier-derived blocks (ISU-3), mélange (ISU-4) as well as basal marine ice (ISU-5) in both along- and cross-flow radargrams; and (iii) demonstrated that simple 1-D models can adequately capture the along-flow evolution suture-zone structures, if constrained by geophysical observations. These developments can provide high-quality constraints for ice-shelf models.

Nonetheless, a range of processes controlling the spatial and temporal evolution of suture zones, their seawater content and temperature and their ability to detain rifts and thus stabilise Antarctic ice shelves must be better understood for their effects to be fully implemented in ice-shelf models. These include for example the provenance, evolution and physical properties of the mélange (ISU-4), the processes that govern basal marine ice (ISU-5) accretion and its along-flow compaction and, most significantly, the processes that control the diffusion of fracture-driving stresses that may eventually allow a rift to break through a suture zone.

## Methods

### Ground-penetrating radar

The two common offset GPR profiles shown here (red dotted arrows in Fig. 2) were acquired with snow-scooter-towed assemblies, using a Sensors & Software PulseEKKO PRO system and 50 MHz antennas^12^. We acquired one trace every 3–4 m at a towing speed of ~12 km h^−1^, representing a distance-averaged stack of eight individual traces at a sampling interval of 0.8 ns. Each stacked trace was located with a handheld GPS linked to the GPR system, achieving a planimetric precision of approximately ±5 m. Velocity–depth profiles were derived from common-midpoint surveys^29^ and used for depth conversion of radargrams.

### Flowline modelling

To place the section we have imaged with GPR into a broader spatial context, we use our established 1-D flowline model^13^ to simulate the zone’s along-flow evolution. We model basal melt and freezing rates (${\dot{\mathrm{b}}}$) from published input data sets of ice-shelf thickness (*Z*) and density (*ρ*)^51^, surface accumulation rate^35^ (${\dot{\mathrm{a}}}$) and the surface velocity vector^52^ (**V**), on the assumption that the mass of the Larsen C Ice Shelf is in steady state (∂ρ**V**Z/∂*t* = 0):

1 $${\dot{\mathrm{b}}} = {\dot{\mathrm{a}}} - \nabla \cdot \left( {{\uprho }}{\mathbf{V}}Z \right)$$

The steady-state assumption is justified for our purposes in that any recent rapid mass imbalance is unlikely to apply to a significant portion of the historical ~300-year timescale of advection from the tip of the JP to location JP-Seis^13^. All data sets are compiled on the same 1 km × 1 km grid in a polar stereographic projection. We adopt a published formulation^20^ to calculate the thicknesses of a surface layer of firn and meteoric accumulated on the ice shelf (FLM-1 in Fig. 5, integrating ISU-1 and ISU-2) and a basal layer of accreted marine ice (FLM-3 in Fig. 5, equivalent to ISU-5) at the downstream edge of each grid node (*Z*_2_) relative to the upstream edge of that node (*Z*_1_):

2 $$Z_2 = Z_1 \ast {\mathrm{e}}^{\dot \varepsilon _z \cdot t} + \frac{{{\dot{\mathrm{a}}}}}{{\dot \varepsilon _z}}\left( {{\mathrm{e}}^{\dot \varepsilon _z \cdot t} - 1} \right)$$

Here *t* is the time taken for the ice to advect between the upstream and downstream edge of the node. The vertical strain rate ($\dot \varepsilon _{\mathrm{z}}$) is calculated as:

3 $$\dot \varepsilon _z = - \left( {\dot \varepsilon _x + \dot \varepsilon _y} \right)$$

where $\dot \varepsilon _{\mathrm{x}}$ and $\dot \varepsilon _{\mathrm{y}}$ are, respectively, the along- and across-flow strain rates calculated from InSAR surface velocities^52^. We initiate the model at the grounding line (Figs. 2 and 3) by assuming that the meteoric ice of the unnamed glacier makes up the full, known^51^, shelf thickness here (FLM-2, equivalent to ISU3 at the grounding line, Fig. 5). At each grid node, we then subtract the cumulative thickness of the surface (FLM-1) and basal (FLM-3) layers calculated using Eq. (2) from known^51^ ice-shelf thickness in that location, yielding an updated thickness for FLM-2. We calculate the thickness of the surface layer from accumulated mass by applying a depth–density profile^53^ constrained by observed firn air content^54^. We calculate the thickness of the basal layer assuming a marine ice porosity of 17%^20^. We can thus partition the full ice thickness at each grid node into a surface layer of accumulated firn and meteoric ice (FLM-1), a central layer of advected glacier ice (FLM-2) and a basal layer of accreted marine ice (FLM-3) (Fig. 5). As explained in the main text and illustrated in Figs. 3 and 4d, the central layer (FLM-2) of advected glacier ice is, in reality, broken into a series of icebergs (ISU-3) separated by rifts filled with mélange (ISU-4). Our model treats the central layer as a continuum of ice with a given thickness at each grid node, irrespective of the provenance of the ice making up that thickness in that location.

### Seismic analysis

Our seismic data were recorded with a *Geometrics Geode* system with *Pentolite* explosive shots as sources, deployed in metre-deep holes drilled with a hand-held auger. Twenty-four spade-deep shallow snow pits were dug and two 100-Hz single-component geophones deployed in each. Of these, one geophone was pushed into the snow at the base of the pit and the second horizontally into the side of it that was transverse to the geophone line and either faced north for the west–east-oriented seismic lines or west for the south–north-oriented lines. Two orthogonal walk-away seismic lines were acquired at JP-Seis and SI-Seis (locations shown in Fig. 2 and example data in Fig. 6). Geophone intervals were 2.5 m over the initial 10 m of offset, 5 m between 10 and 30 m, and 10 m thereafter^55^ up to a maximum source-receiver offset of 1110 m. The survey design thus facilitated both the compressional (P-) and shear (S-) wave imaging of the interior and base of the ice shelf and also the inversion of refraction travel times for depth profiles of density^29^. We established the uncertainty in seismic velocity models (Fig. 7a) using Monte Carlo analysis^56^ (median plus interquartile range) and, using the inverted density model, derived models of the elastic moduli of the glacier-derived meteoric ice block and the basal marine ice beneath it (Fig. 7b). By considering the latter as a two-phase blend of ice and seawater, we investigated plausible ranges of seawater content (Fig. 8) using the H-S bounds^57^:

4 $$K_{{\mathrm{HS}}}^ + = K_1 + \frac{{f_2}}{{\left( {K_2 - K_1} \right)^{ - 1} + f_1\left( {K_1 + \frac{4}{3}{\mathrm{\mu }}_1} \right)^{ - 1}}}$$

5 $${\mathrm{\mu }}_{{\mathrm{HS}}}^ + = {\mathrm{\mu }}_1 + \frac{{f_2}}{{\left( {{\mathrm{\mu }}_2 - {\mathrm{\mu }}_1} \right)^{ - 1} + 2f_1\left( {K_1 + 2{\mathrm{\mu }}_1} \right)/\left[ {5{\mathrm{\mu }}_1/\left( {K_1 + \frac{4}{3}{\mathrm{\mu }}_1} \right)} \right]^{ - 1}}}$$

where $K_{{\mathrm{HS}}}^ +$ and ${\mathrm{\mu }}_{{\mathrm{HS}}}^ +$ are the upper bounds of bulk and shear moduli, in volume fractions *f*_n_, for the bulk (*K*_n_) and shear (*µ*_n_) moduli of the component phases. Equivalent lower bounds, $K_{{\mathrm{HS}}}^ -$ and ${\mathrm{\mu }}_{{\mathrm{HS}}}^ -$, are obtained when indices 1 and 2 are interchanged. The upper and lower bounds of *K* and *µ* describe the strongest and weakest blends of two phases. When one phase is a fluid, the upper bound suggests that the blend is matrix supported, while the lower indicates a fluid-supported suspension. Lying between the H-S bounds is the VRH average^58^; we consider the range of moduli between the VRH and upper H-S bound, thereby assuming that the marine ice is not a fluid-supported suspension of ice crystals. This assumption recognises that the base ice reflection is distinguishable in our seismic data from JP-Seis (Fig. 6c), notwithstanding the fact that this reflection is much weaker than at SI-Seis (Fig. 6b).

### Viscous buckling analysis

By analogy with other geological media^42^, we can relate horizontal wavelengths (*λ*) of observed buckles (Fig. 4a) to the viscosities of the harder in situ meteoric ice layer (*µ*_h_, VBM-2 equivalent to ISU-2 in Fig. 4c, d) and the combined softer units of firn (layer VBM-1, equivalent to ISU-1), mélange (ISU-4), basal marine ice (ISU-5) and glacier-derived blocks (ISU-3) (conglomerate layer VBM-3) (average of *µ*_s_) by:

6 $$\lambda = 2\pi h\root {3} \of {{\frac{{\mu _{\mathrm{h}}}}{{6\mu _{\mathrm{s}}}}}} = \root {3} \of {{\frac{{A_{\mathrm{s}}}}{{6A_{\mathrm{h}}}}}}$$

where *A*_h_ and *A*_s_ are the corresponding rate factors in Glen’s flow law and *h* is the thickness of the harder in situ meteoric ice layer (VBM-2). The harder the VBM-2 layer and the softer the combined layers VBM-1 and VBM-3 (Fig. 4c, d), the larger the amplitudes and wavelengths of buckles formed by deformation. We take four main steps in evaluating the role of temperature (*T*) versus seawater content (*W*) in layer softening. First, we use the Arrhenius relation

7 $$A = A_0{\mathrm{e}}^{ - Q\left( {RT} \right)^{ - 1}}$$

to estimate the rate factor of the in situ meteoric ice layer VBM-2 (*A*_h_), where *R* is the universal gas constant (8.314 J mol^−1^ K^−1^) and *Q* is equal to 139 × 10^3^ J mol^−1^ (*A*_0_ = 3.615 × 10^−13^ s^−1^ Pa^−3^) and 60 × 10^3^ J mol^−1^ (*A*_0_ = 1.733 × 10^3^ s^−1^ Pa^−3^), respectively, for temperatures warmer, or equal to, and colder than −10 °C^22^. For a plausible temperature range of meteoric ice of −5 to −15 °C, as measured on the Larsen C Ice Shelf in the 2014/15 austral summer^23^, *A*_h_ ranges^22^ between 1.6 × 10^−24^ and 2.9 × 10^−25^ s^−1^ Pa^−3^. Second, we measure ranges of in situ meteoric ice thicknesses (*h*, layer VBM-2 in Fig. 4c, d) and horizontal wavelengths (*λ*) of buckles in our GPR data (marked by black arrows in Fig. 4a) and apply Monte Carlo analysis with 10^5^ uniform samples to them and the inferred range of *A*_h_. This yields a range of values for the combined rate factor (*A*_s_) of the softer layers VBM-1 and VBM-3 (Fig. 4c, d) from Eq. (6) (Fig. 9a). Third, we insert these values into Eq. (7) to estimate a possible temperature (*T*) range for these softer layers (Fig. 9b) and, fourth, estimate a possible average range of the seawater content (*W*) for them (Fig. 9c) using^59^

8 $$A_{\mathrm{s}} = A_0\left( {1 + C\,W} \right)$$

where *C* ≈ 181.25 is a constant of proportionality^57^ and *A*_0_ = 2.4 × 10^−24^ s^−1^ Pa^−3^ is the average rate factor of mélange and basal marine ice at −2 °C^22^.
